# Small Molecules Targeting 3C Protease Inhibit FMDV Replication and Exhibit Virucidal Effect in Cell-Based Assays

**DOI:** 10.3390/v15091887

**Published:** 2023-09-06

**Authors:** Sirin Theerawatanasirikul, Varanya Lueangaramkul, Achiraya Pantanam, Natjira Mana, Ploypailin Semkum, Porntippa Lekcharoensuk

**Affiliations:** 1 Department of Anatomy, Faculty of Veterinary Medicine, Kasetsart University, Bangkok 10900, Thailand; 2 Department of Microbiology and Immunology, Faculty of Veterinary Medicine, Kasetsart University, Bangkok 10900, Thailand; varanya.lu@ku.th (V.L.); achiraya.pan@ku.th (A.P.); natjira.ma@ku.th (N.M.); fvetpls@ku.ac.th (P.S.); 3 Center of Advanced Studies in Agriculture and Food, KU Institute, Bangkok 10900, Thailand

**Keywords:** foot-and-mouth disease virus (FMDV), antiviral activity, virtual screening, 3C protease, intracellular protease assay

## Abstract

Foot-and-mouth disease (FMD) is a highly contagious disease in cloven-hoofed animals, caused by the foot-and-mouth disease virus (FMDV). It is endemic in Asia and Africa but spreads sporadically throughout the world, resulting in significant losses in the livestock industry. Effective anti-FMDV therapeutics could be a supportive control strategy. Herein, we utilized computer-aided, structure-based virtual screening to filter lead compounds from the National Cancer Institute (NCI) diversity and mechanical libraries using FMDV 3C protease (3C^pro^) as the target. Seven hit compounds were further examined via cell-based antiviral and intracellular protease assays, in which two compounds (NSC116640 and NSC332670) strongly inhibited FMDV, with EC50 values at the micromolar level of 2.88 µM (SI = 73.15) and 5.92 µM (SI = 11.11), respectively. These compounds could inactivate extracellular virus directly in a virucidal assay by reducing 1.00 to 2.27 log TCID50 of the viral titers in 0–60 min. In addition, the time-of-addition assay revealed that NSC116640 inhibited FMDV at the early stage of infection (0–8 h), while NSC332670 diminished virus titers when added simultaneously at infection (0 h). Both compounds showed good FMDV 3C^pro^ inhibition with IC50 values of 10.85 µM (NSC116640) and 4.21 µM (NSC332670). The molecular docking of the compounds on FMDV 3C^pro^ showed their specific interactions with amino acids in the catalytic triad of FMDV 3C^pro^. Both preferentially reacted with enzymes and proteases in physicochemical and ADME analysis studies. The results revealed two novel small molecules with antiviral activities against FMDV and probably related picornaviruses.

## 1. Introduction

Foot-and-mouth disease is one of the most important viral diseases affecting cloven-hoofed species, including cattle, swine, sheep, goats, and wild species [1,2]. This disease is caused by the foot-and-mouth disease virus (FMDV), which is a member of the *Aphthovirus* genus within the *Picornaviridae* family [3]. FMDV is closely related to and grouped together with significant human pathogenic viruses, such as coxsackievirus (CV), enterovirus (EV), poliovirus (PV), hepatitis A virus (HAV), and human rhinoviruses (HRVs) [4,5]. These picornaviruses cause various diseases, including hand-foot-and-mouth disease, acute poliomyelitis, meningitis, hepatitis, and respiratory infections. There are seven serologically distinct types of FMDV: serotypes A, C, O, and Asia1, and South African Territories (SAT), SAT1, SAT2, and SAT3. Serotypes A, O, Asia I, and SAT1-3 actively circulate in cloven-hoofed animals worldwide, particularly in Asia, Africa, and the Middle East, whereas serotype C has not been reported since 2004 [6,7]. FMDV is highly diverse and contagious, and, thus, it causes major economic losses and seriously affects the livestock industry worldwide. The severity of FMD varies depending on the virus strains and serotypes as well as the affected animal species. The clinical signs of FMD range from mild to severe vesicular formation around the mouth and skin on the feet, nose, muzzle, and teat. Some infected animals show systemic signs, including reduced appetite, weight loss, and reduced milk production.

The prevention and control of FMD are globally regulated by the World Organization for Animal Health [8], but the successful eradication of the disease is still challenging. The culling of infected animals may not be a practical FMDV control measure in some countries. Additionally, there is no cross-protection among different serotypes [2,8]. The transmission of FMD in vaccinated animals can occur even in the absence of clinical signs. Alternative control strategies, such as antiviral agents or small molecules inhibiting FMDV in infected animals, and a serotype-independent vaccine with rapid action are needed [9].

FMDV shares a common characteristic with other picornaviruses, which are small and non-enveloped viruses containing positive-sense, single-stranded RNA genomes of approximately 8.3 kb in length [10]. The genome and virion structures of picornaviruses are highly conserved. The genomic RNA contains a long 5′-untranslated region (UTR), a large single open reading frame (ORF), a short 3′-UTR, and a polyadenylated (poly(A)) tail [4,5]. The viral genome encodes a single polyprotein, which, once translated, is cleaved into four structural and eight non-structural proteins. The structural proteins include VP1, VP2, VP3, and VP4 (also called 1A to 1D) that form the icosahedral capsid during the virion assembly. The non-structural proteins L^pro^, 2A, 2B, 2C, 3A, 3B, 3C protease (3C^pro^), and 3D polymerase (3D^pol^) play important roles in viral biology from RNA replication to viral release [4,5,10]. One of the essential non-structural proteins of picornaviruses, including FMDV, is 3C^pro^. Picornaviral 3C^pro^ has a specific conformation of trypsin-like serine proteases. These proteins possess a Cys-His-Asp/Glu catalytic triad at the active site, in which the arrangement of protein folding resembles the conserved serine proteases [11]. FMDV 3C^pro^ performs 10 of the 13 cleavages of the viral polyprotein and regulates host proteins to facilitate viral replication [12]. Therefore, according to its essential function in the viral life cycle, this enzyme is an attractive target for potential antiviral drug screening.

Extensive studies on the development of picornavirus 3C^pro^ inhibitors have been conducted for HRV, which led to the discovery of AG7088 (also called rupintrivir), a potent irreversible inhibitor, the moiety of which can form a covalent bond with the HRV 3C^pro^ active site [13,14]. Several clinical studies have demonstrated that rupintrivir is not able to reduce the disease severity in natural infections and potentially induce drug resistance upon serial passages of HRV in cells [15,16]. However, rupintrivir has been widely used as a 3C^pro^ inhibitor to demonstrate the direct antiviral action in in vitro studies. Previously, different classes of small-molecular inhibitors against FMDV infection have been identified such as T-1105, an RNA polymerase inhibitor of the influenza virus [17]. T-1105 has also shown proof of concept in reducing FMD clinical signs; however, a high dose of 200–400 mg/kg/day was required for FMD treatment [18], which is not practical for controlling FMDV infections in livestock. Low ranges of nano- to micro-molar concentrations of antiviral molecules are required for large-scale treatment in livestock [9]. High-throughput screening using a computer-aided approach is a useful tool to filter small molecules specific to FMDV 3C^pro^ from the compound libraries. Then, the hit compounds can be further processed and optimized to a promising lead compound for FMD treatment.

In the present study, in silico structure-based virtual screening was performed to predict the small drug-like molecules that would potentially bind to the FMDV 3C^pro^ active site. The filtered inhibitors were examined for their cytotoxicity and inhibitory effects on FMDV replication in cell-based assays. In addition, we evaluated the anti-FMDV 3C^pro^ in a cell-based protease inhibitory assay. Our data provide a starting point for further antiviral drug development against FMDV and other picornaviruses.

## 2. Materials and Methods

### 2.1. Structure-Based Virtual Screening of NCI Compound Libraries

The three-dimensional structure of FMDV 3C protease was modeled from the deduced FMDV 3C^pro^ amino acid sequence (FMDV serotype A; NP05, accession number: MZ923645). 2WV4.pdb was used as the protein template to establish the NP05 3C^pro^ three-dimensional structure according to previous studies [19,20]. The structure of FMDV NP05 3C^pro^ was prepared for the following molecular docking by removing water. Subsequently, by using UCSF Chimera [21], we performed a virtual screening via molecular docking of small molecules in the NCI compound library on the FMDV 3C^pro^ active sites, including the three catalytic residues (His46, Asp84 and Cys163) and the residue that enhances interaction with the substrate (Cys142). The ligand structures were kindly provided by the Developmental Therapeutics Program (DTP) Open Repository of the National Cancer Institute (NCI), for which the ligand libraries consisted of 6409 ligand structures from NCI Diversity set III, VI and V, Mechanical set II, and Mechanical diversity set III. The ligand structures were imported into the Open Babel software toolbox [22] to perform energy minimization. AutoDock Vina [23] embedding in PyRx 0.9.8 virtual screening tool [24] uses a Lamarckian genetic algorithm to generate the binding poses of the ligand inside a protein structure. Hydrogen atoms were added onto the structures of the FMDV 3C^pro^ and ligands, which were converted to pdbqt format. A grid box of size-X = 22, size-Y = 22, and size-Z = 22 was set on the FMDV 3C^pro^, and the grid was centered as center-X = 50, center-Y = 50, and center-Z = 66 [19,25]. The results of virtual screening of ligands and FMDV 3C^pro^ were ranked based on the binding affinity energies of different conformations. The top-ranked compounds were selected from NCI library of the Developmental Therapeutics Program of NCI/NIH and further examined in cell-based assays.

### 2.2. Cells and Viruses

Baby hamster kidney (BHK-21) cells and human embryonic kidney 293T (HEK-293T) cells (ATCC^®^, Manassas, VA, USA) were maintained in a complete medium containing Minimum Essential Medium (MEM, Invitrogen^TM^, Carlsbad, CA, USA), 10% fetal bovine serum (FBS, Invitrogen^TM^, Carlsbad, CA, USA), 2 mM L-glutamine (Invitrogen^TM^, Carlsbad, CA, USA), and 1 × Antibiotic-Antimycotic (Invitrogen^TM^, Carlsbad, CA, USA) at 37 °C with 5% CO_2_. The BHK-21 cells were used for viral propagation and evaluation of intracellular antiviral effect, whereas HEK-293T cells were used for determining cell cytotoxicity and the intracellular protease activity as well as the transfection of plasmids containing FMDV 3C^pro^.

FMDV serotype A (NP05) was propagated five times in BHK-21 cells as previously described [19,25]. The supernatant containing the virus was collected, and viral titers were determined via the Reed and Muench method [26] and reported as 50% tissue culture infectious dose (TCID50). The virus stock with a titer of 1 × 10^9^ TCID50/mL was stored at −80 °C in aliquots for each single use. All experiments associated with live FMDV were conducted in a BSL-2 laboratory with enhanced facilities.

### 2.3. Cytotoxicity Assay

The top-ranked compounds from the virtual screening data were kindly provided by the Developmental Therapeutics Program (DTP) Open Repository of the National Cancer Institute (NCI). Compounds were dissolved in dimethyl sulfoxide (DMSO) as 10 mM stock solutions and stored at −80 °C until use. For cytotoxicity assay, the compound treatment was evaluated in BHK-21 and HEK-293T cells. Briefly, compound stocks were serially diluted to concentrations of 250, 200, 100, 50, 25, 10, 5, 1, and 0.1 µM in MEM without serum. The cells were seeded in a 96-well plate at 2.0 × 10^4^ cells per well and incubated at 37 °C with 5% CO_2_ overnight. The next day, the confluent monolayer of BHK-21 and HEK-293T cells was incubated with the indicated concentrations of the tested compounds in a total volume of 100 µL/well at 37 °C with 5% CO_2_ for 24 h. After incubation, 20 µL of MTS solution (CellTiter 96^®^ Aqueous One Solution Cell Proliferation Assay, Promega, Madison, WI, USA) was added to the cells. The formazan color reaction was measured at 490 nm using a multi-mode reader (Synergy H1 Hybrid Multi-Mode Reader, BioTek^®^, Winooski, VT, USA) after 2 h of cell incubation at 37 °C. The cytotoxicity of the tested compounds was examined in both BHK-21 and HEK-293T cells. The cell viability was analyzed using the following formula as recommended by the manufacturer (Promega, Madison, WI, USA).

[OD treated − OD cell control]/[OD dmso − OD cell control] × 100(1)

### 2.4. Antiviral Activity

Since the tested compounds in this study were selected via virtual screening based on the FMDV NP05 3C^pro^ structure, we sought to elucidate the effects of compounds during virus entry. To accomplish this, three treatment assays were initially performed as follows (Figure S1).

#### 2.4.1. Post Entry Assay

BHK-21 cells were seeded at 2.0 × 10^4^ cells/well in 96-well plates and were grown overnight. On the experiment day, the cells were inoculated with FMDV at 10 TCID50/well. At 2 h after incubation, the cells were washed before incubating with the serially diluted compounds at 37 °C with 5% CO_2_ for 24 h.

#### 2.4.2. Co-Treatment Assay

To determine the viral inactivation effects of compounds against FMDV before infection, BHK-21 cells were seeded overnight at 2.0 × 10^4^ cells/well in a 96-well plate and incubated with FMDV at 10 TCID50/well and the serially diluted compounds at 37 °C with 5% CO_2._ After incubation for 2 h, the unabsorbed virus was removed, and then the cells were washed with PBS twice. The cells were maintained in MEM supplemented with 2% FBS at 37 °C with 5% CO_2_ for 24 h.

#### 2.4.3. Pre-Treatment Assay

To determine whether the compounds could protect the cells from viral infection, the overnight-grown BHK-21 cells in a 96-well plate were pretreated with the compounds at 37 °C with 5% CO_2_ for 2 h, followed by viral infection of the pre-treated cells for another 2 h. Subsequently, the cells were washed twice with PBS. The cells were then maintained in MEM supplemented with 2% FBS at 37 °C with 5% CO_2_ for 24 h.

Once the compounds demonstrated antiviral activity in the co-treatment and the post-entry assays, four additional assays were performed as follows.

#### 2.4.4. Attachment Assay

BHK-21 cells seeded in 96-well plates at 2.0 × 10^4^ cells/well were incubated overnight. The next day, BHK-21 cells were pre-chilled on a plate at 4 °C for 1 h, and then each tested compound and FMDV (10 TCID50/well) were incubated with the pre-chilled cells at 4 °C for 1 h to allow for virus attachment. After incubation, the supernatant with unbounded virus was removed, and the cells were washed once with PBS and incubated with MEM containing 2% FBS at 37 °C with 5% CO_2_ for 24 h.

#### 2.4.5. Penetration Assay

The overnight-seeded BHK-21 cells in 96-well plates were pre-chilled by incubating at 4 °C for 1 h. Subsequently, the cells were incubated with FMDV (10 TCID50/well) at 4 °C for another hour. Then, the inoculum was removed, and the cells were washed with PBS to eliminate the unattached viruses. The serially diluted compounds were then added into the culture media, and the plates were transferred to 37 °C with 5% CO_2_ to allow virus penetration into the cells for 2 h. The cells were then washed with PBS, overlaid with MEM supplemented with 2% FBS, and incubated at 37 °C with 5% CO_2_ for 24 h.

Once the incubation times of the attachment and penetration assays were complete, the reduction in FMDV-infected cells by the compounds at each concentration in each experimental assay was determined through an immunostaining assay (Immunoperoxidase monolayer assay, IPMA) to evaluate the half-maximal effective concentration (EC50) against virus infection. DMSO at 0.1% was used as a negative drug control, while rupintrivir was used as a positive drug control.

#### 2.4.6. Virucidal Activity (Time Course Assays)

To investigate the virucidal effects of compounds exhibiting antiviral activity in the co-treatment assay, we conducted a series of experiments. Initially, 10 TCID50 of FMDV was directly incubated with the maximum non-toxic dose (MNTD90) of the tested compounds, as determined via the cytotoxicity assay, in 1.5 mL microtubes. These virus–compound mixtures were incubated at 37 °C for 0, 15, 30, and 60 min. Subsequently, the remaining viruses after the compound treatments were titrated as follows. The virus–compound mixtures were ten-fold serially diluted in MEM from 10^−1^ to 10^−7^ before adding 100 µL of each dilution to each well of the overnight-seeded BHK-21 cells in 96-well plates. The cells were then incubated at 37 °C with 5% CO_2_ for 72 h. Subsequently, the infected cells in each well were observed and recorded, and the virus titers were calculated using Reed and Muench method [26]. The resulting data were presented as log TCID50/mL, enabling a comprehensive evaluation of the virucidal effects of the compounds across the selected concentration range.

#### 2.4.7. Time-of-Addition (ToA) Assay

The compounds that could inhibit FMDV in the post-entry assay were further tested for the inhibition stage of FMDV life cycle. The BHK-21 cells were seeded at 2.0 × 10^5^ cells/well in 24-well plates and incubated overnight. The compounds were added before (−2 h), during (0 h), and after infection at +2, +4, +6, and +8 h in separate wells. At the time after infection, the inoculum was incubated with the cells to allow for virus absorption for 2 h, and then the unabsorbed viruses were removed. The cells were then washed with PBS thrice, before adding the fresh MEM media with 2% FBS and incubating at 37 °C with 5% CO_2_. Then, the compounds were added into the media at different time points (2–8 h), and the cells were incubated for 10 h post-infection. The cells and supernatant were harvested separately to collect both intra- and extracellular viruses, and viral RNA was determined using RT-qPCR.

### 2.5. Immunoperoxidase Monolayer Assay (IPMA)

To determine the viral infection in the BHK-21 cells, IPMA was performed following an established method elsewhere [27]. Briefly, the cells were fixed with 150 µL cold methanol for 20 min and washed twice with 1 × PBS with tween 20. Then, the cells were incubated with 2% BSA in PBS, followed by 3% hydrogen peroxide in DW. Subsequently, the cells were washed and incubated with a single-chain variable fragment with Fc fusion protein (scFv-Fc) specific to 3ABC of FMDV (dilution at 1:3000) at 37 °C for 1 h for viral antigen detection. After incubation, the cells were washed and then incubated with protein G and HRP conjugate (dilution 1:1000, EMD Millipore corporation, Temecula, CA, USA) at 37 °C for 1 h. To evaluate the viral antigens in the infected cells, the cells were visualized by using DAB substrate (DAKO, Santa Clara, CA, USA) to develop dark-brown color of FMDV-infected cells. The samples were observed and imaged by using a phase-contrast inverted microscope (Olympus IX73, Tokyo, Japan). All experiments were repeated at least twice.

### 2.6. Determination of FMDV Copy Number Using RT-qPCR

BHK-21 cells were seeded in 24-well plates at a density of 2.0 × 10^5^ cells/well and incubated at 37 °C overnight. The effective compounds against FMDV infection from antiviral activity assay were carried out for viral load quantification using RT-qPCR. Either intracellular or extracellular viruses were harvested at 24 h post-infection. The total RNAs were isolated from both infected cells and supernatant separately using Trizol reagent (Thermo Fisher Scientific, Waltham, MA, USA), combined with Direct-zol^TM^ RNA MiniPrep (Zymo Research Corporation, Tustin, CA, USA) following the manufacturer’s instruction. RNA concentration was determined with a NanoDrop^TM^ 2000c Spectrophotometer (Thermo Fisher Scientific, Waltham, MA, USA). cDNA synthesis was then performed with RevertAid reverse transcriptase (Thermo Fisher Scientific, Waltham, MA, USA) according to the manufacturer’s instruction. Briefly, equal amounts of the total RNAs (1 µg) from the infected cells treated with different concentrations of the compounds and from the virus control were mixed to 4 µL of random hexamers (Invitrogen^TM^, Carlsbad, CA, USA) and RNase-free ddH_2_O to a final volume of 12.5 µL. The reaction mixtures were incubated at 25 °C for 10 min. After incubation, 4 µL of 5 × reaction buffer, 2 µL of 10 mM dNTP, 0.5 µL of 20 UI RiboLock RNase Inhibitor (Thermo Fisher Scientific, Waltham, MA, USA), and 1 µL of 200 U RevertAid reverse transcriptase (Thermo Fisher Scientific, Waltham, MA, USA) were added to the reaction tube before addition of RNase-free ddH_2_O to a final volume of 20 µL. Then, the mixture was incubated at 42 °C for 60 min, followed by 70 °C for 10 min. Then, 2 µL of cDNA was used as the templates in the qPCR using iTaq Universal SYBR Green Supermix (Bio-Rad Laboratories, Hercule, CA, USA) with 0.5 µL of each primer specific to the FMDV 5′UTR, fwd primer: 5′-CTGTTGCTTCGTAGCGGAGC-3′, and rev primer: 5′-TCGCGTGTTACCTCGGGGTACC-3′, and RNase-free ddH_2_O to a final volume of 10 µL, as previously described [28]. The qPCR amplifications were conducted after first denaturation at 95 °C for 30 s, composed of 40 cycles of denaturation at 95 °C for 5 s and annealing/extension step at 60 °C for 30 s. A melting curve was analyzed following the default setting of the CFX96 touch Real-Time PCR detection system (Bio-Rad Laboratories, Hercule, CA, USA). The viral copy numbers of samples were determined using the absolute quantification method. To generate a standard curve, plasmids containing FMDV 5′UTR were ten-fold serially diluted from 10 to 10^−5^ plasmid molecules/µL for viral load quantification. All experiments were repeated at least twice with two biological duplicates. The data are presented as the means ± standard deviations (SDs) of viral copy numbers and percentages of viral reductions relative to that of virus control.

### 2.7. Intracellular Protease Assay

To determine the inhibitory effect on FMDV 3C^pro^ activity, intracellular protease assay was modified from the previously published method [29] described elsewhere [19,25]. This assay comprises three essential components, including a plasmid containing pBV_3ABCD as FMDV 3C^pro^ positive control or pBV_mu3ABCD as FMDV 3C^pro^ negative control [19] and pG5Luc (Promega, Madison, WI, USA). pBV_3ABCD contains an intact FMDV 3C protease-coding region, while 3C^pro^ with Cys142Ser and Cys163Gly generated from the previous study [30] was the inserted sequence in pBV_mu3ABCD. Both 3ABCD and mu3ABCD sequences in plasmids pBV were flanked by a Gal4-binding domain coding sequence at 5′ and a VP16 activation domain coding sequence at 3′. pG5Luc is a reporter plasmid containing the Gal4-sequence upstream of the firefly luciferase gene under the control of the VP16. The pBV_3ABCD and pBV_mu3ABCD also contained *Renilla* luciferase gene downstream the 3ABCD and mu3ABCD sequences, which served as an internal control.

To analyze the intracellular protease, HEK-293T cells in Opti-MEM (Gibco^TM^ Thermo Fisher Scientific Inc., Waltham, MA, USA) were seeded in a 96-well plate at 2 × 10^5^ cells/well and incubated at 37 °C with 5% CO_2_ overnight. The cells were co-transfected with 100 ng/well of a plasmid containing pBV_3ABCD or pBV_mu3ABCD and 100 ng/well of pG5Luc (Promega, Madison, WI, USA). The plasmid DNAs were transfected using Fugene^®^ HD (Promega, Madison, WI, USA) at 0.6 µL per µg DNA. The transfection of each treatment and control was duplicated. After two hours of transfection, the cells were treated with serially diluted compounds or 0.1% DMSO in Opti-MEM. DMSO and rupintrivir (anti-3C^pro^ drug) were included as negative and positive drug controls, respectively. After compound treatment, the cells were incubated at 37 °C for 16 h, and the transfected media were removed. The cells were then washed with 1× PBS and lysed using 20 µL of Passive Lysis Buffer (Promega, Madison, WI, USA). The signals of firefly and *Renilla* luciferase activities were then determined using the Dual-Glo Luciferase Assay System (Promega, Madison, WI, USA), and the signal intensity affected by the compounds was recorded using Synergy H1 Hybrid Multi-Mode Microplate Reader (BioTek, Winooski, VT, USA). The full spectra of luciferase signals were generated when the compound completely inhibited 3C protease, resulting in binding of GAL4 to the Gal4-binding domain and guiding the VP16 activation domain in proximity of VP16 to drive the luciferase gene expression. The data were presented as a ratio of firefly/*Renilla* luminescent signal (Fluc/Rluc) from the compound-treated wells, which was compared to the signals obtained from wells transfected with pBV_mu3ABCD (3C^pro^ negative plasmid control, also generated the full spectra of luciferase activity).

### 2.8. In Silico Molecular Interaction and Physicochemical Properties of Compounds

The predicted FMDV 3C^pro^-ligand complexes were analyzed using Discovery Studio Visualizer, version 2021 (BIOVIA, Dassault Systèmes, San Diego, CA, USA) and UCSF Chimera, version 1.16 (UCSF, San Francisco, CA, USA) to reveal their interactions, such as hydrogen bonding, π-π interaction, π-sigma, and π-cation interactions between ligand and FMDV 3C^pro^ protein. The candidate compounds were analyzed for their physicochemical characteristics and the ADME properties, including absorption, distribution, metabolism, and excretion parameters, using SWISS-ADME tool predictor [31] and DataWarrior software version 5.5.0 [32]. SwissTargetPrediction [33] was performed to explore the biological and chemical targets of the candidate compounds.

### 2.9. Statistical Analysis

The cytotoxicity results were analyzed and reported as the half-maximal cytotoxic concentration (CC50), which is the concentration of the compound that reduces the cell viability by 50%, whereas the maximum non-toxic dose (MNTD90) refers to as the dose at which the cell viability is at least 90%. Data were calculated with non-linear regression method using GraphPad Software version 9.4.1 (Prism, San Diego, CA, USA). 

To increase the antiviral assay performance, Z′ factor analysis was used to normalize the discrepancy of the signals between plates. The Z′ factor was calculated using the following equation.

1 − [(3 × standard deviation of the cell control)^i^ + (3 × standard deviation of the virus control)^i^/ (mean of the cell control^ii^ − mean of the virus control^ii^)] (2)

where the superscripts i and ii are cell and virus controls from different plates. The Z′ factor that was equal to or greater than 0.5 indicates a sensitive and reliable evaluation of this assay [34]. The half-maximal effective concentration (EC50) of each compound was determined as 50% viral reduction in FMDV infection by the compounds compared to that by 0.1% DMSO (set to 100%), which was analyzed and fit to the curves using a non-linear regression in GraphPad Software version 9.4.1 (Prism, San Diego, CA, USA). Values were not extrapolated if they were greater than concentrations of the tested range. The half-maximum inhibitory concentration (IC50) of compound against protease activity refers to the 50% inhibitory concentration required to inhibit the intracellular FMDV 3C^pro^. The IC50 was obtained from intracellular protease assays. The selective index (SI) was calculated:

SI = CC_50_/EC_50_ (3)

## 3. Results

### 3.1. Structure-Based Virtual Screening of Small Molecules Using AutoDock Vina

A virtual screening approach was used to filter biologically active small molecules belonging to the NCI compound libraries containing 6409 compounds that bound to the FMDV 3C^pro^ structure. Upon screening of the NCI’s library, we identified the seven top-ranked compounds that potentially exhibited promising anti-FMDV 3C^pro^ activity. The physicochemical properties following Lipinski’s (L5 rule) [35] and ADME analysis of the initial hit compounds are presented in Table 1 and Figure S2. The seven hit compounds with good binding affinities ranging from −6.6 to −6.8 kcal/mol were then further examined in the cell-based antiviral assay.

### 3.2. Antiviral Activity against FMDV Infection

The seven hit compounds were initially tested in BHK-21 cell lines to determine their cytotoxicity. The CC50 values of the seven compounds varied, as shown in Table 2. Once the compounds passed the cytotoxicity assay, we then evaluated their antiviral activities in the BHK-21 cells by using dose ranges between CC50 and MNTD90. The mechanism of antiviral activity against FMDV was divided into three main stages, designated as “post-viral entry”, “co-treatment”, and “pre-treatment”, and the antiviral activity of the seven hit compounds was measured in relation to the reduction in positive infected cells using IPMA.

**Figure 1:**
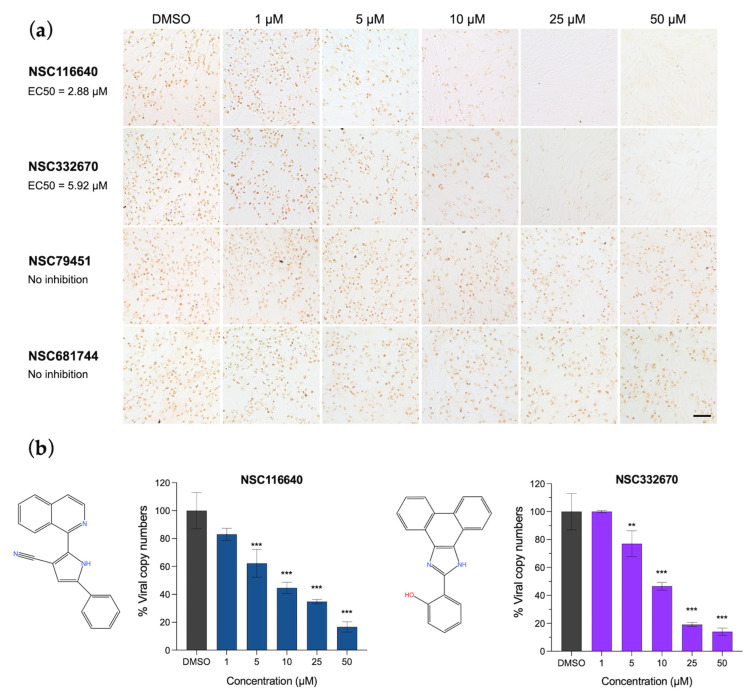
Antiviral activities of NSC116640, NSC332670, NSC79451, and NSC681744 in a dose-dependent manner. (**a**) FMDV antigen detection using IPMA to elucidate the ability of NSC116640 and NSC332670 to inhibit FMDV replication in the post-viral entry stage. (**b**) Quantification of viral copy number from FMDV-infected BHK-21 cells after treatment with NSC116640 and NSC332670 at the indicated concentrations using RT-qPCR. The 0.1% DMSO was used as negative control. The data are presented as mean and SD. *** *p* < 0.001; ** *p* < 0.01.

In the post-viral entry, the results were evaluated when the cells were treated with the compounds after they were infected by FMDV. Compounds NSC116640 and NSC332670 could inhibit FMDV replication with an EC50 of 2.88 ± 0.17 and 5.92 ± 0.20 µM, respectively (Figure 1a). Thus, these two compounds were selected as the leads for further investigation. We also evaluated the viral load reduction using RT-qPCR. Both compounds could reduce the viral loads in a dose-dependent manner, which corresponded to the finding in the immunostaining evaluation (Figure 1b). The SI values of these two compounds were 73.15 for NSC116640 and 11.11 for NSC332670. The five remaining compounds demonstrated a low inhibition potency at the post-viral entry stage.

**Figure 2:**
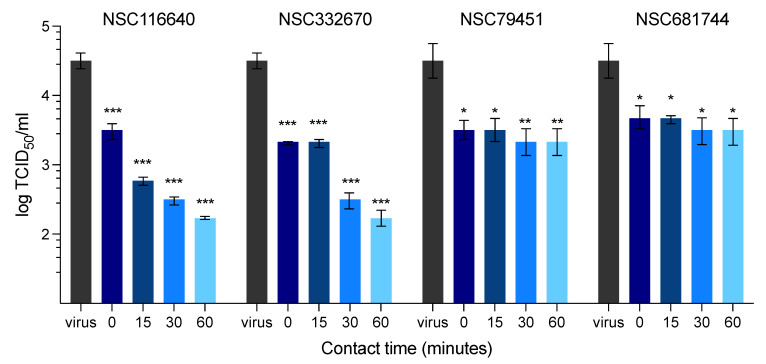
Evaluation of the virucidal effect of compounds at different time points (0, 15, 30, and 60 min) on FMDV replication over 72 h present as log TCID50. The data are presented as means and SD. *** *p* < 0.001; ** *p* < 0.01; * *p* < 0.05.

For the co-treatment assay, the compound treatment and FMDV infection took place simultaneously. Four of seven compounds showed a potent inhibitory effect during FMDV infection (Table 2), as they could protect cells from viral infection, which was evidenced by the reduction in the positive infected cells in the IPMA (Figure S3). To elucidate the exact inhibitory mechanism of the selected compounds, we further tested the ability of the compounds to inhibit FMDV at different stages of infection, including during viral binding (attachment) and viral penetration to the host cells. The results revealed that none of the compounds showed antiviral effects during viral attachment and penetration. Thus, the compounds might be permeant so that they could pass the cell membrane to protect the cells from viral infection during co-incubation with the virus for 24 h. These results were in accordance with the prophylactic effect of the two selected compounds in the pre-treatment assay (NSC116640 with EC50 of 7.27 ± 0.86 µM and NSC332670 with EC50 of 7.18 ± 0.98 µM), as they could protect the cells and reduced the cytopathic effect caused by viral infection. Moreover, the compounds that showed good antiviral activity in the co-treatment assay were further examined in the virucidal assay at different timepoints. Our selected compounds, NSC116640 and NSC332670, at 50 µM, significantly reduced the number of live and infectious viruses as they could reduce the viral titers by 1.00–2.27 log TCID50/mL and 1.17–2.27 log TCID50/mL, respectively. Thus, both compounds significantly killed the viruses at 0 min and had a greater virucidal effect up to 60 min. Both NSC79451 and NSC681744 at 50 µM demonstrated a good inhibition at 0 min, but their virucidal actions did not increase by the incubation times (from 0 to 60 min) (Figure 2).

**Figure 3:**
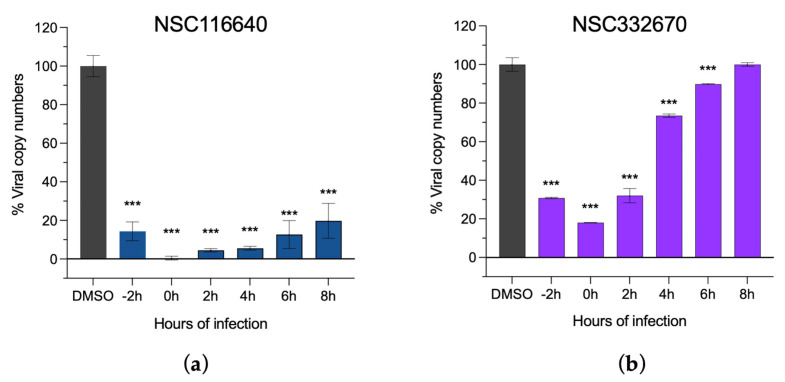
Time-of-addition assay of (**a**) NSC116640 and (**b**) NSC332670 demonstrating viral inhibition of the compounds. The compounds were added before (−2 h), during (0 h), and after viral infection from +2 to +8 h, and virus control was treated with 0.1% DMSO (DMSO). Viral copy numbers were evaluated using RT-qPCR. The data are presented as means and SD. *** *p* < 0.001.

In addition, the ToA assay was used to validate the effects of the two compounds at each stage of viral infection (NSC116640 and NSC332670), which were incubated with the cells at an MNTD90 dose of 50 µM. The ToA result (Figure 3) revealed that NSC116640 could reduce virus yield (0–10% of viral copy numbers) when treating the cells between −2 and +4 h post-infection (hpi) but the number of viruses was slightly increased when treated at +6 and +8 hpi (Figure 3a). NSC332670 showed a great inhibition when treated before (−2 h), during (0 h), and after viral infection (+2 h). The best inhibition of NSC332670 was at 0 h, which was at the same time of virus infection. However, its inhibition effect was reduced when treated after infection at +4 and +6 h while completely lost at +8 h post-infection (Figure 3b).

### 3.3. Intracellular FMDV Protease Inhibition Assay

**Figure 4:**
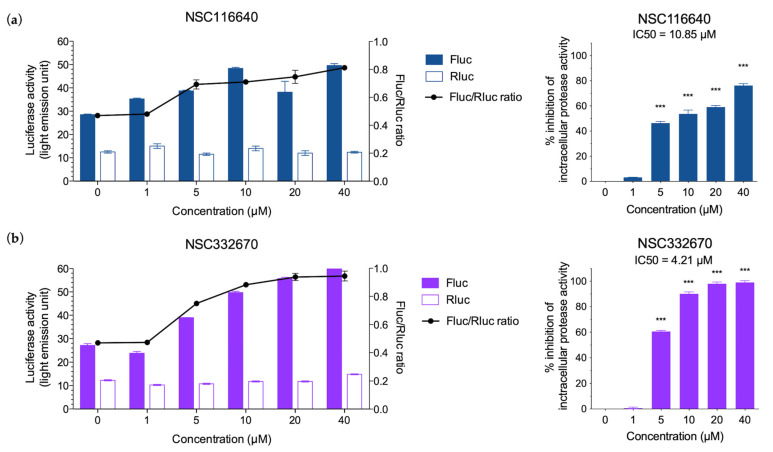
Intracellular protease assay of (**a**) NSC116640 and (**b**) NSC332670. Fold increases in FLuc/Rluc ratios in the HEK-293T cells transiently expressed with FMDV 3C^pro^ compared to that of 0.01% DMSO as a negative drug control. Filled color and empty bars are luciferase activities from firefly (Fluc) and *Renilla* (Rluc) luciferase, respectively. Inhibitory effects of the compounds on intracellular FMDV 3C^pro^ activity appear in a dose-dependent manner. The data are presented as means and SD. *** *p* < 0.001.

Two compounds (NSC116640 and NSC332670) that presented a good antiviral activity at the early-stage post-viral infection were further tested in the intracellular protease assay. We used serially diluted compounds up to the MNTD90 dose of 50 µM, which was not toxic to HEK-293T cells. The results of intracellular protease inhibition are shown as the fold increase in FLuc/RLuc ratio, in the presence of the transient expression of FMDV 3C^pro^ in HEK-293T cells. NSC116640 and NSC332670 could inhibit the protease activity in a dose-dependent manner (Figure 4). NSC332670 inhibited FMDV 3C^pro^ (IC50 = 4.21 µM) nearly 90% at 10 µM and 100% at 40 µM, while NSC116640 (IC50 = 10.85 µM) required more than 40 µM for 80% inhibition compared to 0.1% DMSO control. The IC50 values of NSC116640 and NSC332670 for FMDV 3C^pro^ inhibition were 10.85 and 4.21 µM, respectively.

### 3.4. Protein–Ligand Interactions and Target Prediction of Candidate Compounds

**Figure 5:**
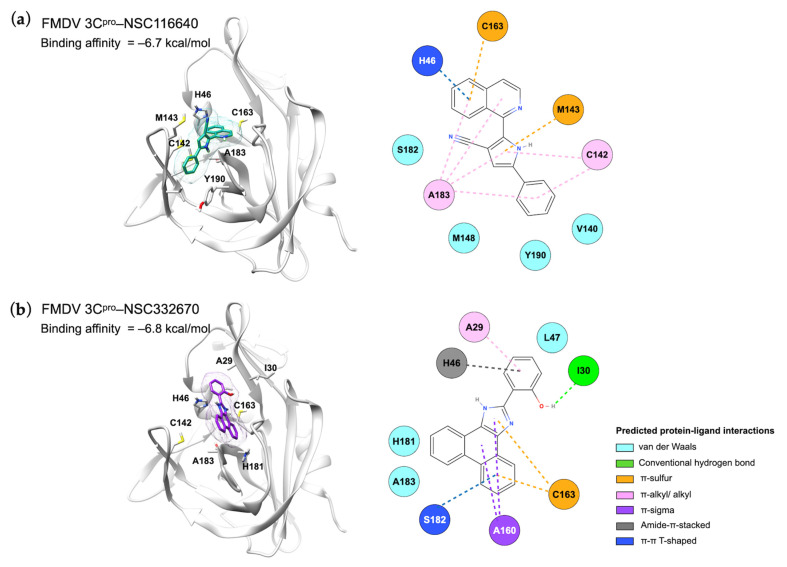
Molecular interactions between FMDV 3C^pro^ and ligands (**a**) NSC116640 and (**b**) NSC332670 displayed in the 3D (**left**) and 2D (**right**) images.

Our study showed that two candidate compounds inhibited FMDV infection and targeted FMDV 3C^pro^, as shown in the intracellular FMDV protease inhibition assay (Figure 4). We evaluated the protein–ligand interaction by focusing on the active site of FMDV 3C^pro^. The prediction model demonstrated that the compounds reacted to the active residues of 3C^pro^. The isoquinoline core structure of NSC116640 interacted with the catalytic residues, which are His46 with π-π T-shaped interaction and Cys163 by π-sulfur interaction. In addition, the pyrrole ring—a heterocyclic aromatic structure—of the compound reacted to Cys142 of 3C^pro^, which stabilized the compound via π-alkyl interaction. The dihydrophenanthrene and imidazol-2-ylidene structures of NSC332670 could react to Cys163 with π-sulfur interaction, whereas the cyclohexa-2,4-dien-1-one bound to His46 via amide-π-stacked interaction. However, the compounds did not reach the Asp84 in the depths of the 3C^pro^ binding pocket. The 2D and 3D structures of protein–ligand interactions of both candidate compounds with FMDV 3C^pro^ are shown in Figure 5. In addition, we evaluated the compound structure to estimate the most probable targets of both candidate compounds using in silico prediction, which relied on bioactivity data from the ChEMBL database [36]. The target prediction analysis of both candidate compounds revealed the top 15 target classes, for which the major targets of NSC116640 were predicted to be enzyme (26.7%) and protease (13.3%), whereas those of NSC332670 were kinase (33.3%), enzyme (26.7%), and protease (13.3%) (Figure S4). The in silico predicted data corresponded to our studies that appeared to directly inhibit FMDV 3C^pro^.

## 4. Discussion

3C protease (3C^pro^) is a conserved viral enzyme of picornaviruses. This protein could serve as a common virulence factor, which not only contributed to the vital role in the viral life cycle but also the modulation of the host proteins for virus replications [11,37]. The FMDV 3C^pro^ structure has an architecture homology to a classical trypsin-fold structure of picornaviral 3C^pro^, which contains two six-strand β-barrels connected perpendicularly to each other. The surface cleft between two β-barrels forms a binding pocket, which contributes to substrate recognition of the active site [38,39]. The catalytic triad in the active site of FMDV 3C^pro^ consists of Cys163, His46, and Asp84. Additionally, Cys142 of FMDV 3C^pro^ forms a flap to strengthen the substrate stability, which is crucial for proteolytic activity [38,39]. Therefore, the picornaviral 3C^pro^ has essential roles in the cleavage of the viral polyprotein for maturation of the structural and the non-structural proteins. In addition, picornaviral 3C^pro^ also cleaves the host cellular factors to promote viral RNA replication by hijacking the host cell gene regulation and expression machinery [10,11,37]. It has been reported that FMDV 3C^pro^ switched cellular proteins to induce viral RNA replication through the cleavage of cellular translation-associated proteins, e.g., translation initiation factors eIF4A [40] and the 68 kDa Src associated in mitosis protein (SAM68) [41]. Regarding the certain functions of 3C^pro^, this protein is one of the most attractive targets for the antiviral drugs for picornaviruses or related viruses, including FMDV. In this study, the compound libraries were filtered to find active small molecules against the 3C^pro^ of FMDV, using various approaches, such as protein-based and cell-based assays. Another high-throughput assay was an in silico assay, which could predict the potential interaction of the lead molecules that bind to the active site on the target enzyme. We used a combinational screening workflow comprising both structure-based virtual screening and antiviral cell-based assays to select for 3C^pro^-targeting compounds with anti-FMDV action. Moreover, the specific target of the selected compounds was evaluated using the intracellular 3C^pro^ inhibitory assay, which confirmed the actual function of the lead molecules in the host cells. Herein, we found two out of four compounds (NSC116640 and NSC332670) with potential anti-FMDV by targeting 3C^pro^.

NSC116640 is a 2-isoquinolin-1-yl-5-phenyl-1H-pyrrole-3-carbonitrile, which contains isoquinoline and pyrrole rings as the major scaffolds. Our molecular docking and physicochemical analysis predicted the interaction of the lead compounds and the enzyme. It was found that two residues in the catalytic triad (His46 and Cys163), and the Cys142 residue at the tip of the β-ribbon close to the active site played an important role, similar to L127 of HRV 3C^pro^ [42]. In addition to the protease activity, Cys142 has been shown to alter the host type-I interferon (IFN) responses via RIG-I and MDA5 degradation [43]. This prediction revealed that this compound might block or interfere with substrate recognition of FMDV 3C^pro^ and the enzyme function. NSC116640 has been developed as an anti-cancer agent, which inhibits a cellular FADD-like interleukin-1β-converting enzyme inhibitory protein (c-FLIP) as a selective c-FLIP inhibitor. This compound actively reduces c-FLIP/FADD interaction and modulates caspase-8/10 activation in the apoptotic pathway [44]. It has been suggested that blocking the c-FLIP inhibitor (e.g., LBH589) might be beneficial to reduce viral replication at the early stage of SARS-CoV-2 infection in COVID-19 patients (in the review by [45]). Moreover, NSC116640 has previously been screened for anti-FMDV using StopGo (glycline/proline) luciferase activity (PubChem AID: 1159524, 2015) with potent activity at 28.18 µM.

NSC332670 (6-(1,3-Dihydrophenanthro[9,10-d]imidazol-2-ylidene)cyclohexa-2,4-dien-1-one) is another lead compound against FMDV 3C^pro^ in our study. This compound has been tested for antimicrobial and anti-cancer activities. NSC332670 has been confirmed to reduce carboxypeptidase B activity with an IC50 value of 1.55 mM. This protease enzyme is important for *Plasmodium* spp. development in the midgut of mosquitoes [46]. In addition, this compound has been shown to be able to actively inhibit human respiratory syncytial virus (RSV) with a CC50 value of 15.27 µM (PubChem AID: 2391, 2010) and an IC50 value of 10.95 µM (PubChem AID: 2410, 2010) in HEP-2 cells. However, the target protein of RSV for antiviral activity has not been investigated. Herein, we demonstrated that these two compounds could inhibit FMDV 3C^pro^ intracellularly with the potent micromolar levels and had virucidal effects on FMDV in a timepoint-dependent manner. In the case of NSC116640 and NSC332670, it is possible that they could be potential multitarget agents for FMDV.

While NSC116640 and NSC332670 exhibited promising anti-FMDV potential, it is important to note that these findings stem from an in vitro study using optimal concentrations of both viruses and compounds. Such carefully selected concentrations were utilized to facilitate a clear observation of the inhibitory effects. However, the micromolar concentrations at which these compounds manifest antiviral activity may not necessarily translate to effective dosages when administered to animals. In addition, the predictive interaction between the compounds and 3C^pro^ should be established through co-crystallization of the compounds and recombinant 3C^pro^. This process will provide a more concrete confirmation of the molecular interactions at play. Despite these considerations, our study corroborates prior research, indicating the inherent antiviral properties of NSC116640 and NSC332670 [45, PubChem AID: 1159524, 2015; PubChem AID: 2410, 2010].

NSC79451 (2-hydroxy-N-(2-methyl-5-nitrophenyl)-3-nitrobenzamide) and NSC681744 (N-[6-[benzoyl-[cyano(phenyl)methyl]amino]hexyl]-N-[cyano(phenyl)methyl]benzamide) are diverse chemical compounds in the mechanical diversity sets of the NCI library. Both NSC79451 and NSC681744 were investigated using high-throughput yeast halo assays; however, none of them could inhibit *Saccharomyces cerevisiae* and *Schizosaccharomyces pombe* [47]. Additionally, no antiviral activity or application of these two compounds has been reported thus far. Our study found that both compounds could reduce extracellular viruses in the in vitro virucidal assay. They possessed less gastrointestinal absorption and brain penetration, as confirmed through the BOILED-Egg prediction [48].

## 5. Conclusions

Conclusively, FMD is a picornavirus that infects cloven-hoof animals and causes severe losses in livestock production worldwide. The disease widely spreads in endemic areas where vaccination is implemented. Effective antiviral agents for FMD control are needed to minimize the shed virus and protect the infected animals from clinical signs. This study discovered two novel chemical compounds targeting FMDV 3C^pro^, which could inhibit FMDV infection in vitro. NSC116640 and NSC332670 presented promising results as anti-FMDV candidates based on our biological assay and in silico computational data, which could pave the way for further antiviral development.

## Figures and Tables

**Table 1:** The initial hit compounds using structure-based virtual screening in this study. Compounds demonstrated by the NCI ID, IUPAC name, the 2D structure, and physicochemical parameters of drug likeness based on Lipinski’s 5 rules.

NCI ID and IUPAC Name	Structure	Molecular Weight (g/mol)	H-Bond Donor Count	H-Bond Acceptor Count	Rotatable Bond Count	Log-*P*	TSPA (Å)
NSC116640 2-isoquinolin-1-yl-5-phenyl-1H-pyrrole-3-carbonitrile (Binding affinity = −6.7 kcal/mol)	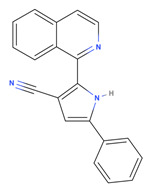	295.34	1	2	2	4.769	52.47
NSC332670 2-isoquinolin-1-yl-5-phenyl-1H-pyrrole-3-carbonitrile (Binding affinity = −6.7 kcal/mol)	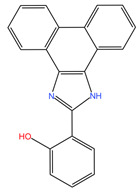	310.35	2	2	1	5.242	48.91
NSC79451 2-Hydroxy-N-(2-methyl-5-nitrophenyl)-3-nitrobenzamide (Binding affinity = −6.6 kcal/mol)	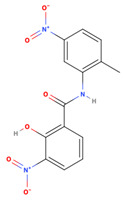	317.25	2	6	5	2.769	140.97
NSC681744 N-[6-[benzoyl-[cyano(phenyl)methyl]amino]hexyl]-N-[cyano(phenyl)methyl]benzamide (Binding affinity = −6.7 kcal/mol)	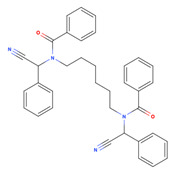	554.68	0	4	15	7.361	88.2
NSC64672 8-[(2E)-2-[(4-hydroxy-3-methoxyphenyl)methylidene]hydrazinyl]-1,3-dimethyl-7H-purine-2,6-dione (Binding affinity = −6.7 kcal/mol)	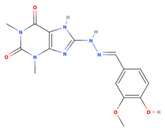	334.33	3	9	4	0.12	126.53
NSC119805 2-[(E)-[(E)-[(E)-3-(2-nitrophenyl)prop-2-enylidene]hydrazinylidene] methyl]phenol (Binding affinity = −6.7 kcal/mol)	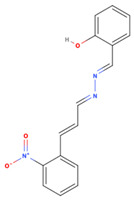	295.29	1	5	5	3.419	88.09
NSC331757 N-[5-(diethylamino)pentan-2-yl]-9,10-dioxoanthracene-2-carboxamide;hydrochloride (Binding affinity = −6.8 kcal/mol)	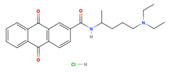	428.96	1	4	9	4.124	66.48
Reference values (Lipinski’s rule [35])		<500	<5	<10	<10	<5	<140

**Table 2:** The CC50 and EC50 values of compounds with binding energy ranging from −6.6 to −6.8 kcal/mol.

Compounds	CC50 (µM) ^1^	EC50 (µM) ^2^		
		Post Entry	Co-Treatment	Pre-Treatment
NSC116640	210.70 ± 0.17	2.88 ± 0.17	5.74 ± 0.23	7.27 ± 0.86
NSC332670	65.82 ± 0.06	5.92 ± 0.20	2.04 ± 0.02	7.18 ± 0.98
NSC79451	32.79 ± 0.3	I	1.56 ± 0.03	I
NSC681744	124.00 ± 0.5	I	4.83 ± 0.08	I
NSC64672	130.09 ± 2.30	I	I	I
NSC119805	82.45 ± 0.40	I	I	I
NSC331757	62.78 ± 0.87	I	I	I

^1^ The half-maximal concentrations of compounds, which were toxic to BHK-21 cells as determined by MTS assay. ^2^ The half-maximal effective concentrations of compounds, which inhibit FMDV infection in BHK-21 cells as determined by IPMA. I: Inactive.

## Data Availability

No new data were created or analyzed in this study. Data are available upon requested.

## Supplementary Materials

The following supporting information can be downloaded at: https://www.mdpi.com/article/10.3390/v15091887/s1, Figure S1: The experimental design of antiviral activity studies. Figure S2: Structure-based virtual screening of NCI repository collection library. (**a**) The binding affinity of the compounds in each library. (**b**) The BOILED-Egg prediction of the compounds depicts gastrointestinal absorption and brain penetration. BBB (yellow zone; blood–brain barrier) and HIA (white zone; human intestinal absorption). Blue dot (PGP+) and red dot (PGP−) represent positive and negative biochemical efflux (e.g., P-glycoprotein pumping out substrates from central nervous system (CNS)), respectively. (**c**) The initial hit compounds were selected for further cell-based assay, in which the predicted physicochemical space presents the pink color zone as for oral bioavailability. LIPO (lopophility: −0.7 < XLOGP3 < +5.0; SIZE: 1560 g/mol < MV < 500 g/mol; POLAR (polarity): 20Å^2^ < TPSA < 130 Å^2^; INSOLU (insolubility): −6 < LogS (ESOL) < 0; INSATU (instaturation): 0.25 < Fraction Csp3 < 1; FLEX (flexibility): 0 < Number rotatable bonds < 9. Figure S3: The antiviral activity of co-treatment assay which the serially diluted compounds were co-incubated with FMDV (10 TCID50/well). Scale bar is 100 µM. Figure S4: Target prediction of compounds (**a**) NSC116640 and (**b**) NSC332670.
