# Supplementary material for: Small Molecules Targeting 3C Protease Inhibit FMDV Replication and Exhibit Virucidal Effect in Cell-Based Assays
Source: Viruses. 2023 Sep 6;15(9):1887. doi: 10.3390/v15091887 (PMC10535379; doi:10.3390/v15091887)
Supplement: Supplementary file 1 [file viruses-15-01887-s001.zip › viruses-2572061-supplementary.pdf]

Supplementary Materials

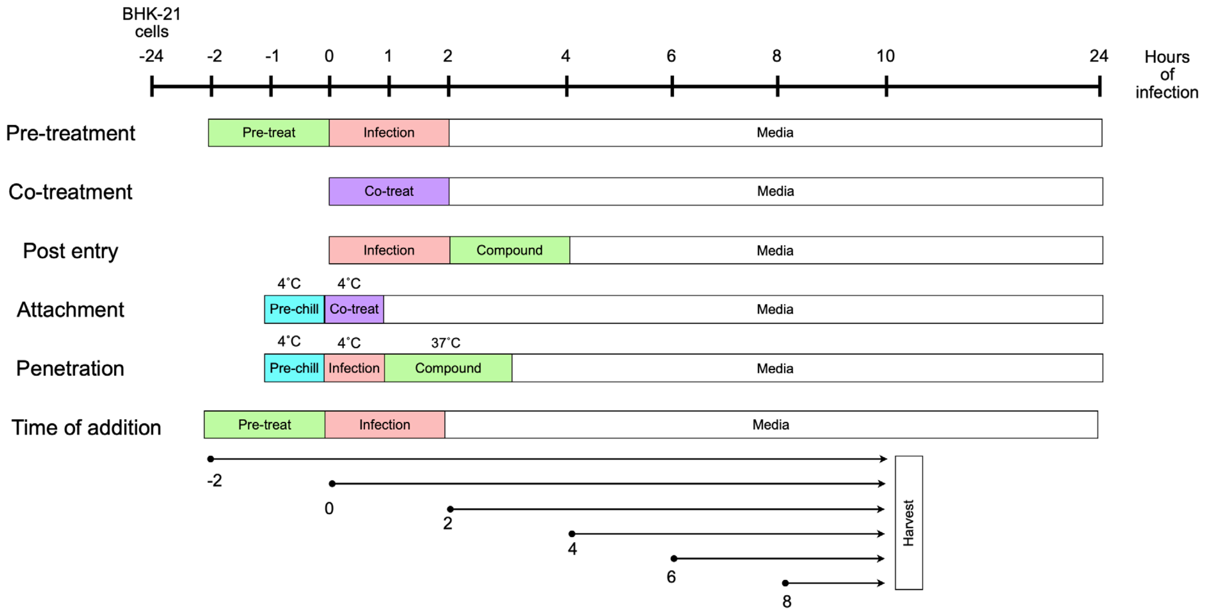

Figure S1. The experimental design of antiviral activity studies.

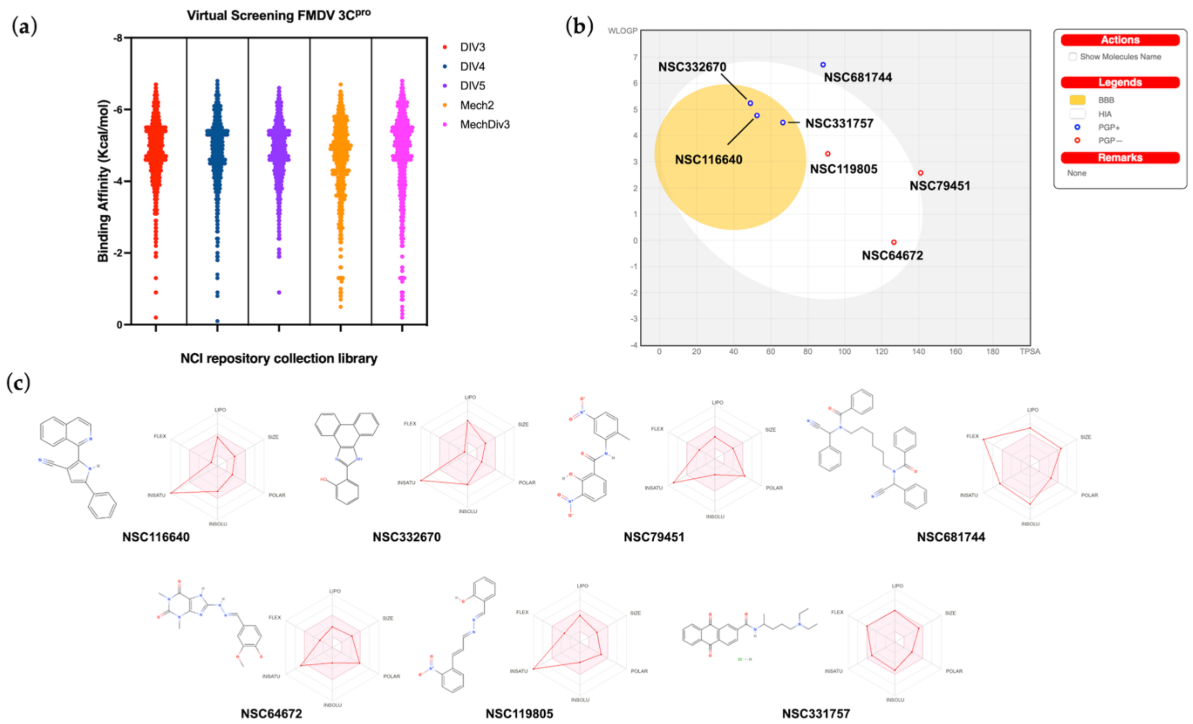

Figure S2. Structure-based virtual screening of NCI repository collection library. (**a**)The binding affinity of the compounds in each library. (**b**) The BOILED-Egg prediction of the compounds depicts the gastrointestinal absorption and brain penetration. BBB (yellow zone; blood-brain barrier) and HIA (white zone; human intestinal absorption). Blue dot (PGP+) and red dot (PGP−) represent positive and negative biochemical efflux (e.g., P-glycoprotein pumping out substrates from central nervous system (CNS)), respectively. (**c**) The initial hit compounds were selected for further cell-based assay, which the predicted physicochemical space presents the pink color zone as for oral bioavailability. LIPO (lopophility: -0.7 < XLOGP3 < +5.0; SIZE:1560g/mol < MV < 500g/mol; POLAR (polarity): 20$Å^2$ < TPSA < 130 $Å^2$; INSOLU (insolubility): -6 < LogS (ESOL) < 0; INSATU (instaturation): 0.25 < Fraction Csp3 < 1; FLEX (flexibility): 0 < Number rotatable bonds < 9.

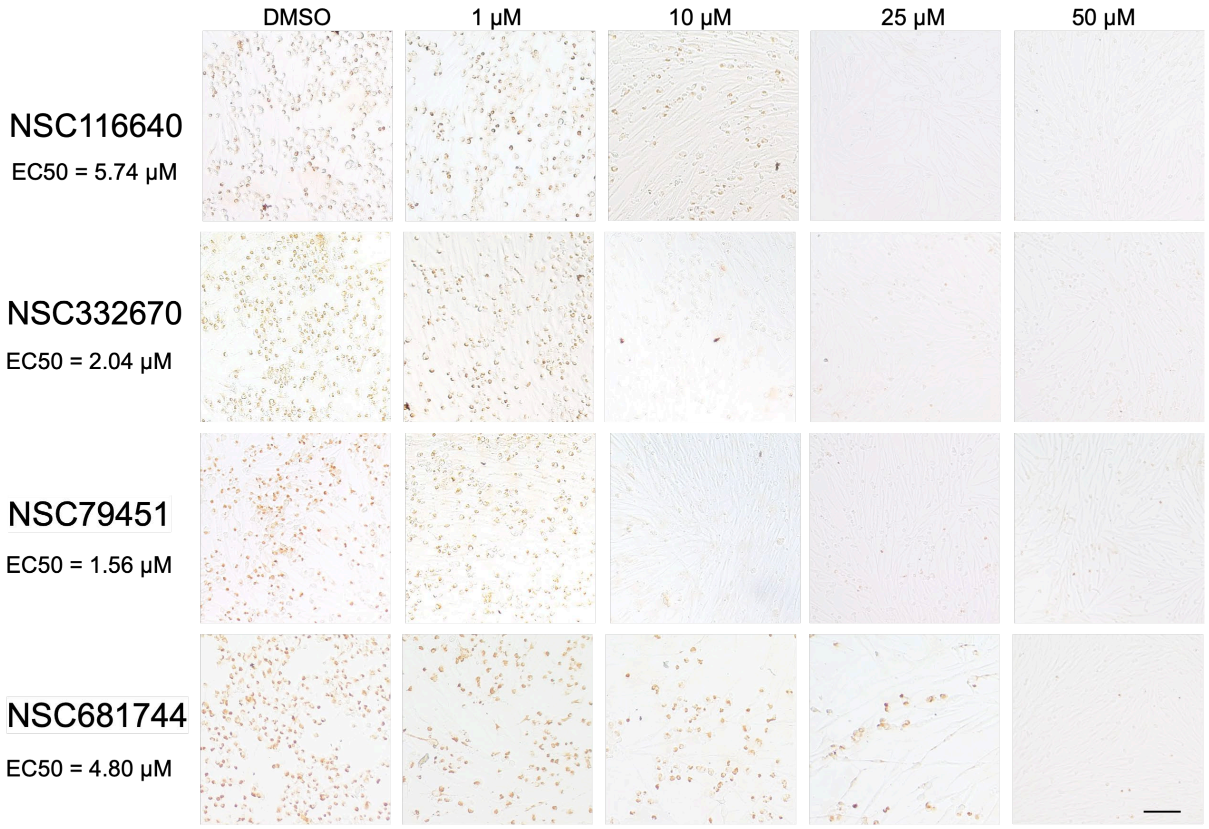

Figure S3. The antiviral activity of co-treatment assay which the serially diluted compounds were co-incubated with FMDV (10 TCID50/well). Scale bar is 100 μM.

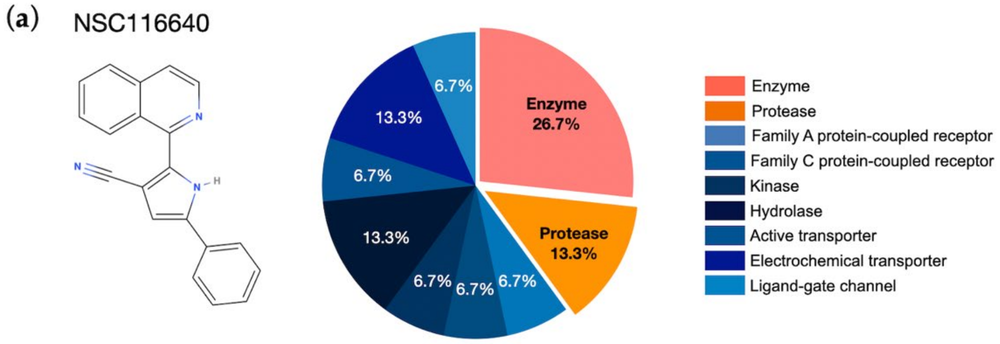

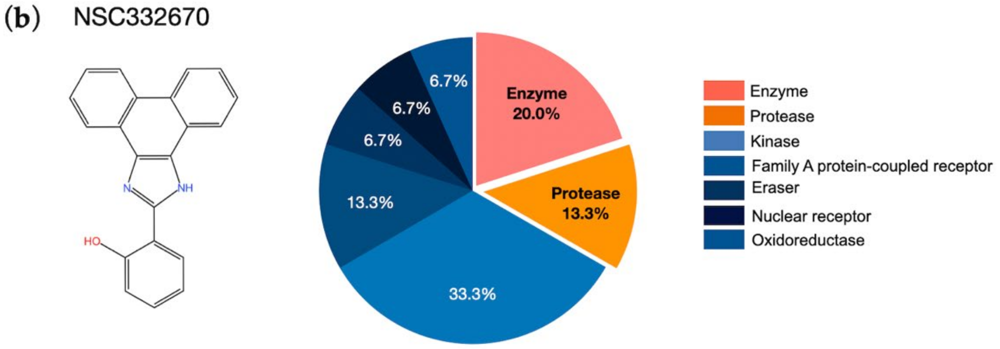

Figure S4. Target prediction of compounds (**a**) NSC116640 and (**b**) NSC332670.
